# Comparative genomic analysis of the *Growth-Regulating Factors-Interacting Factors* (*GIFs*) in six Salicaceae species and functional analysis of *PeGIF3* reveals their regulatory role in *Populus* heteromorphic leaves

**DOI:** 10.1186/s12864-024-10221-5

**Published:** 2024-03-28

**Authors:** Yuqi Yang, Jianhao Sun, Chen Qiu, Peipei Jiao, Houling Wang, Zhihua Wu, Zhijun Li

**Affiliations:** 1 Key Laboratory of Biological Resource Protection and Utilization of Tarim Basin, Xinjiang Production and Construction Group, 843300 Alar, China; 2 College of Life Science and Technology, Tarim University, 843300 Alar, China; 3 Desert Poplar Research Center of Tarim University, 843300 Alar, China; 4 College of Biological Sciences and Technology, Beijing Forestry University, 100083 Beijing, China; 5 College of Life Sciences, Zhejiang Normal University, 321004 Jinhua, China

**Keywords:** Salicaceae, *Populus Euphratica*, *GIF* genes, Heteromorphic leaves, Regulatory function

## Abstract

**Background:**

The *growth-regulating factor-interacting factor* (*GIF)* gene family plays a vital role in regulating plant growth and development, particularly in controlling leaf, seed, and root meristem homeostasis. However, the regulatory mechanism of heteromorphic leaves by *GIF* genes in *Populus euphratica* as an important adaptative trait of heteromorphic leaves in response to desert environment remains unknown.

**Results:**

This study aimed to identify and characterize the *GIF* genes in *P. euphratica* and other five Salicaceae species to investigate their role in regulating heteromorphic leaf development. A total of 27 *GIF* genes were identified and characterized across six Salicaceae species (*P. euphratica*, *Populus pruinose*, *Populus deltoides*, *Populus trichocarpa*, *Salix sinopurpurea*, and *Salix suchowensis*) at the genome-wide level. Comparative genomic analysis among these species suggested that the expansion of *GIFs* may be derived from the specific Salicaceae whole-genome duplication event after their divergence from *Arabidopsis thaliana*. Furthermore, the expression data of *PeGIFs* in heteromorphic leaves, combined with functional information on *GIF* genes in *Arabidopsis*, indicated the role of *PeGIFs* in regulating the leaf development of *P. euphratica*, especially *PeGIFs* containing several *cis*-acting elements associated with plant growth and development. By heterologous expression of the *PeGIF3* gene in wild-type plants (Col-0) and *atgif1* mutant of *A. thaliana*, a significant difference in leaf expansion along the medial-lateral axis, and an increased number of leaf cells, were observed between the overexpressed plants and the wild type.

**Conclusion:**

*PeGIF3* enhances leaf cell proliferation, thereby resulting in the expansion of the central-lateral region of the leaf. The findings not only provide global insights into the evolutionary features of Salicaceae *GIFs* but also reveal the regulatory mechanism of *PeGIF3* in heteromorphic leaves of *P. euphratica*.

**Supplementary Information:**

The online version contains supplementary material available at 10.1186/s12864-024-10221-5.

## Background

Salicaceae species are commonly chosen as exemplary forest trees in various research studies owing to their ease of vitro regeneration, rapid vegetative reproduction, and significant ecological and economic importance across the Northern Hemisphere [1–4]. In particular, *Populus euphratica* characterized by its extraordinary tolerance to drought and salinity, serves as a pivotal species in desert oases and stands out as an exceptional relic plant thriving in extremely arid environments [5, 6]. *P. euphratica* exhibits a distinct pattern of leaf heteroblasty, with adult trees displaying a sequential production of linear (Li, leaf index ≥ 4), lanceolate (La, 2 ≤ leaf index < 4), ovate (Ov, 1 ≤ leaf index < 2), and broad ovate (Bo, leaf index < 1) leaves as they ascend from the lower canopies to the upper canopies, accompanied by a gradual increase in leaf width and area [7]. Previous research indicated that broad Ov and Bo leaves in *P. euphratica* exhibit greater resilience to drought than narrow Li and La leaves, as evidenced by their thicker palisade tissue and enhanced photosynthetic activity [8]. In addition, a significant and positive association was observed between leaf area and the content of proline [9]. So, the presence of heteromorphic leaves in *P. euphratica* suggests its capacity to adjust to the dry desert environment. Hence, identifying and characterizing the genes involving in the heteromorphic leaf development of *P. euphratica* are crucial for revealing the functional divergence and adaptive evolution of heteromorphic leaves.

*Growth-regulating factors-Interacting Factors* (*GIFs*) represent a class of transcriptional co-activators that collaborate with *growth-regulating factors* (*GRFs*). Typically, *GIFs* can interact with *GRFs*, forming a plant-specific transcriptional complex [10]. The *GIF* gene family was initially discovered in *Arabidopsis thaliana* in 2004 [11]. *GIF* genes have been intricately linked with plant growth and development [12, 13]. In a recent study, it was revealed that *GIFs* play a vital role in maintaining the precise expression patterns of key developmental factors [14]. *GIF* transcriptional coregulators assume the responsibility of regulating the quiescent center organization and the meristem size in *A. thaliana* [15]. *GIF2* and *GIF3* are two additional proteins instrumental in cell proliferation and the development of lateral organs [16]. G*if2* and *gif3* mutations have been associated with the production of smaller lateral organs than the wild-type plant species in *A. thaliana* [17, 18]. The single *gif* mutant lines in *A. thaliana* presented a phenotype akin to the control, whereas the *gif* triple mutant *gif1/gif2/gif3* exhibited an aberrant pistil [18].

Beyond their roles in model plants, *GIFs* exert considerable influence in rice [19, 20], maize [21], tomato [22], and tea [23]. Although *GIFs* have been investigated in certain plant species, further exploration of this gene family is warranted in Salicaceae. Recent advancements in the genomics of Salicaceae species offer an opportunity to characterize the *GIF* gene family. In the presents research, a genome-wide analysis of the *GIF* gene family was conducted. The structures, conserved motifs, *cis*-elements, and expression patterns of this family were characterized, a comparative genomics analysis was performed across six different Salicaceae species. The role of *PeGIF3* in the heteromorphic leaf development in *P. euphratica* was further revealed through heterologous expression in *A. thaliana*. The results could offer crucial and precious data for forthcoming investigations concerning the functional characterization of the *GIF* gene family in Salicaceae species.

## Results

### Identification and characterization of *GIFs* in six Salicaceae species

**Fig. 1:**
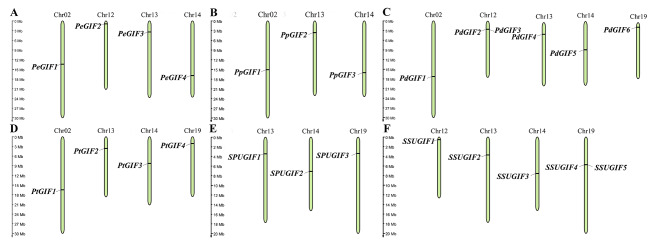
Chromosomal distribution of *GIF* genes across six Salicaceae species. **(A)** *P. euphratica*, **(B)** *P. pruinose*, **(C)** *P. deltoides*, **(D)** *P. trichocarpa*, **(E)** *S. sinopurpurea*, (F) *S. suchowensis*

A total of four, four, six, four, four, and five *GIF* genes were identified in *P. euphratica*, *Populus pruinose*, *Populus deltoides*, *Populus trichocarpa*, *Salix sinopurpurea*, and *Salix suchowensis*, respectively. Then, in accordance to the location on chromosomes, the *GIF* members within the Salicaceae species were assigned names as follows: *PeGIF1*-*4*, *PpGIF1*-*4*, *PdGIF1*-*6*, *PtGIF1*-*4*, *SPUGIF1*-*4*, and *SSUGIF1*-*5*. Almost all of the *GIF* genes were located on single chromosome in the six Salicaceae species (Fig. 1). Subsequently, the characteristics of *GIF* genes and their encoded proteins were comprehensively analyzed (Table 1). The analysis of protein sequences revealed that the GIF proteins have the potential to encode amino acids ranging from 79 to 223, with a molecular weight varying between 8930.16 and 23495.28 kDa. Additionally, their isoelectric point was observed to fall within the range of 4.71–5.93.

**Table 1:** Characteristics of the putative *GIF* genes

Gene name	Gene ID	Location	Protein length (aa)	Molecular weight (kDa)	Isoelectric point
*PeGIF1*	PeuTF02G01597	Chr02:12874304–12877465	208	22222.92	5.86
*PeGIF2*	PeuTF12G00083	Chr12:839394–841554	145	16772.92	5.48
*PeGIF3*	PeuTF13G00452	Chr13:3281496–3286828	222	23467.23	5.84
*PeGIF4*	PeuTF14G00928	Chr14:16293026–16296957	208	21936.71	5.71
*PpGIF1*	PprTF02G1616	Chr02:13813899–13817581	207	22171.92	5.8
*PpGIF2*	PprTF13G0375	Chr13:3286491–3293070	222	23467.23	5.84
*PpGIF3*	PprTF14G0788	Chr14:14661389–14664180	208	21918.74	5.71
*PpGIF4*	PprTF001Sca0483	Scaffold_1:12022707–12025799	145	16772.92	5.48
*PdGIF1*	Podel.02G196800	Chr02:15354515–15358211	208	22168.95	5.76
*PdGIF2*	Podel.12G027600	Chr12:2249106–2252488	79	8930.16	4.71
*PdGIF3*	Podel.12G028600	Chr12:2321321–2324421	145	16772.92	5.48
*PdGIF4*	Podel.13G046400	Chr13:327323–3278180	222	23495.28	5.84
*PdGIF5*	Podel.14G107200	Chr14:7854454–7858065	208	21909.73	5.71
*PdGIF6*	Podel.19G012500	Chr19:1302084–1307547	203	21240.66	5.65
*PtGIF1*	Potri.002G177600	Chr02:13823330–13823716	208	22207.01	5.76
*PtGIF2*	Potri.013G043700	Chr13:3030096–3035620	222	23495.28	5.84
*PtGIF3*	Potri.014G103900	Chr14:6958276–6961644	208	21940.74	5.71
*PtGIF4*	Potri.019G013100	Chr19:1780203–1785830	220	23318.92	5.72
*SPUGIF1*	Sapur.013G040600	Chr13:2904809–2909490	223	23476.13	5.93
*SPUGIF2*	Sapur.014G081800	Chr14:5980851–5984234	207	21852.55	5.56
*SPUGIF3*	Sapur.019G021900	Chr19:2819423–2824004	221	23451.06	5.72
*SPUGIF4*	Sapur.T169700	Scaffold_634:4046–7420	207	21852.55	5.56
*SSUGIF1*	EVM0027484	chr12:427650–430279	144	16652.72	5.48
*SSUGIF2*	EVM0003176	chr13:3108857–3113513	223	23462.11	5.93
*SSUGIF3*	EVM0005186	chr14:6392169–6395843	207	21894.63	5.57
*SSUGIF4*	EVM0014238	chr19:4835538–4839405	221	23421.03	5.72
*SSUGIF5*	EVM0030974	chr19:4862151–4866018	211	22321.82	5.72

### Analysis of phylogenetic and conserved motifs in multi-species of *GIFs*

**Fig. 2:**
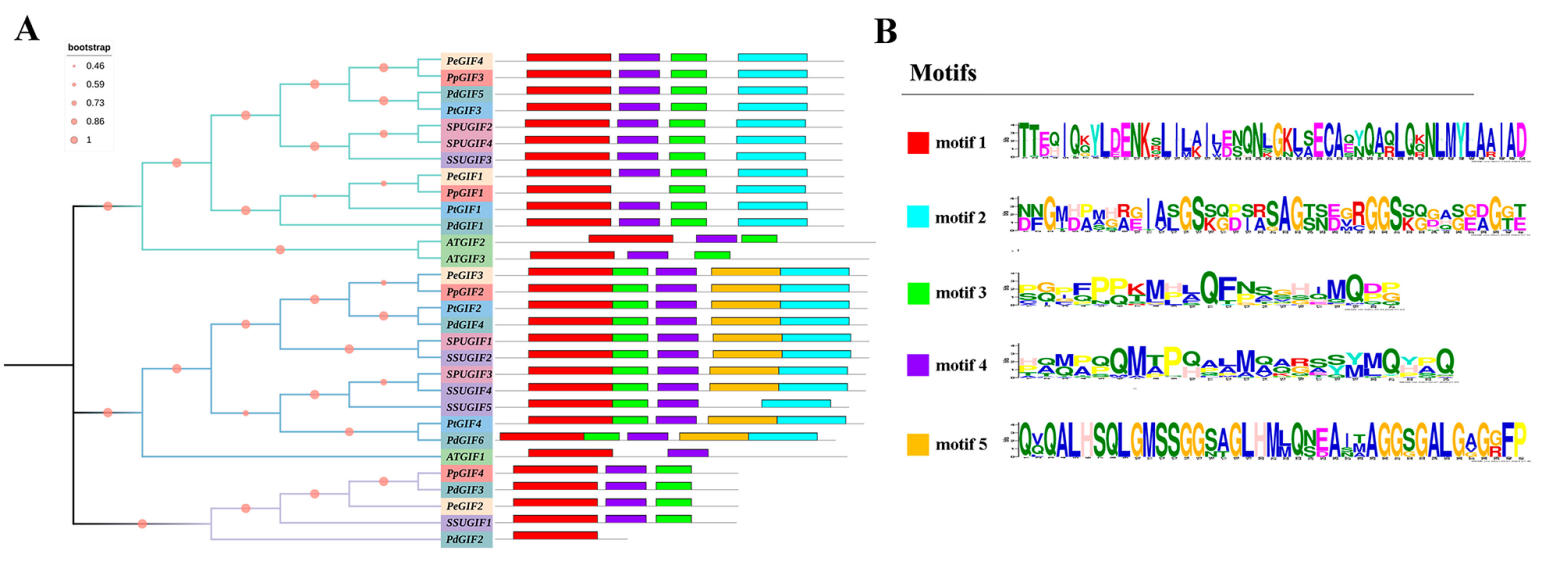
Analysis of conserved motifs and phylogenetic relationships among *GIF* genes across seven species. **(A)** Neighbor-joining (NJ) phylogenetic tree between *P. euphratica* and other six species followed by conserved motifs. The three groups were marked with different colors on tree branches. **(B)** The 30 *GIF* genes in seven species have 5 conserved motifs

The evolutionary relationships and potential functions of 30 *GIFs* from six Salicaceae and one Brassicaceae species were investigated by constructing a phylogenetic tree. The analysis confirmed the classification of *GIF* genes into three subfamilies, denoted as group I-III. Specifically, group I encompassed 13 genes (two *ATGIFs*, two *PeGIFs*, two *PpGIFs*, two *PdGIFs*, two *PtGIFs*, two *SPUGIFs*, and *SSUGIF3*), group II comprised 12 genes (*ATGIF1*, *PeGIF3*, *PpGIF2*, two *PtGIFs*, two *PdGIFs*, two *SPUGIFs*, and three *SSUGIFs*), group III contained five genes (two *PdGIFs*, *PpGIF4*, *PeGIF2*, and *SSUGIF1*) uniquely belonged to Salicaceae (Fig. 2A). The unique *GIFs* from group III indicated they occurred after that divergence between *Arabidopsis* and Salicaceae. Then, MEME software was employed to predict the conserved motifs in these *GIF* genes. Five motifs were identified, with motif 1 representing the conserved SSXT (SNH) domain located in the *N*-terminal region, common among most *GIF* genes. Notably, motif 3 and motif 4 were present in most genes, whereas motif 2 exclusively appeared in group I and II genes. Motif 5 was primarily found in group II members, suggesting its uniqueness to this group. Motifs 2 and 5 at C-terminus were presented in the *GIF* members of Salicaceae species only, possibly indicating their unique roles. Therefore, besides the expanded gene number of *GIFs* in Salicaceae, the variations of GIF domain numbers may also contribute to the novelty of *GIFs* in Salicaceae.

### Collinearity analysis of multi-species *GIFs*

**Fig. 3:**
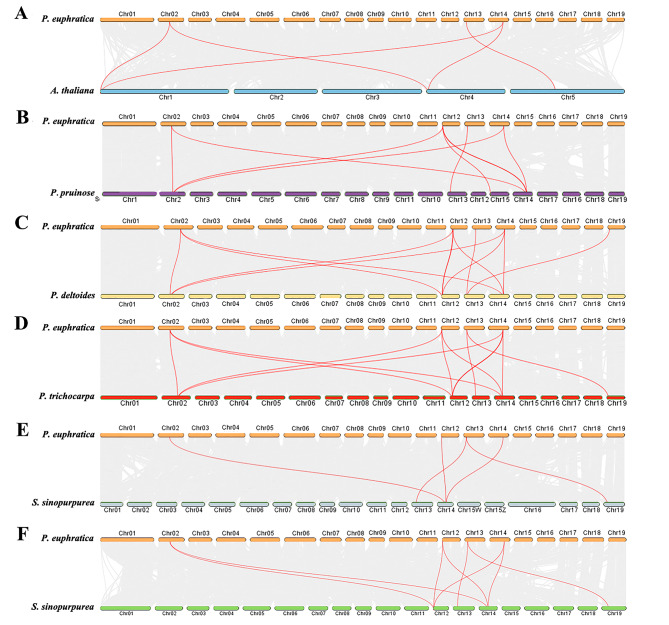
Analysis of collinearity among *GIFs* involving *P. euphratica* and six additional species. **(A)** *P. euphratica* and *A. thaliana*, **(B)** *P. euphratica* and *P. pruinose*, **(C)** *P. euphratica* and *P. deltoides*, **(D)** *P. euphratica* and *P. trichocarpa*, **(E)** *P. euphratica* and *S. sinopurpurea*, **(F)** *P. euphratica* and *S. suchowensis*. The presence of gray lines in the background denotes collinear blocks within *P. euphratica* and other plant genomes, and the red lines emphasize collinear *GIF* pairs

A collinear analysis of *GIFs* between *P. euphratica* and other six species (*A. thaliana, P. pruinose*, *P. deltoides*, *P. trichocarpa*, *S. sinopurpurea* and *S. suchowensis*) was conducted to further investigate the evolutionary processes of *PeGIFs* in *Populus*. The number of *GIF* collinear fragments between *P. euphratica* and the other species were as follows: eight pairs with *P. pruinose* and *S. suchowensis*, twelve pairs with *P. deltoides* and *P. trichocarpa*, and five pairs with *S. sinopurpurea*, respectively (Fig. 3). This result revealed that the collinearity of *GIFs* within the poplar species was more conservative than that between *P. euphratica* and *A. thaliana* (five collinear fragments). These results suggested that the *GIF* gene family is relatively conserved without dramatic expansion nor loss in Salicaceae. Notably, 50 pairs of *PeGIF* genes exhibited collinear relationships between *P. euphratica* and the other five Salicaceae species, indicating that these *GIF-*included collinear fragments likely predated the ancestral divergence. The retention of *GIF-*included collinear fragments possibly resulted from the whole genome duplication that occurred in Salicaceae [24].

### Analysis of *cis*-regulatory elements of *GIF* Genes

**Fig. 4:**
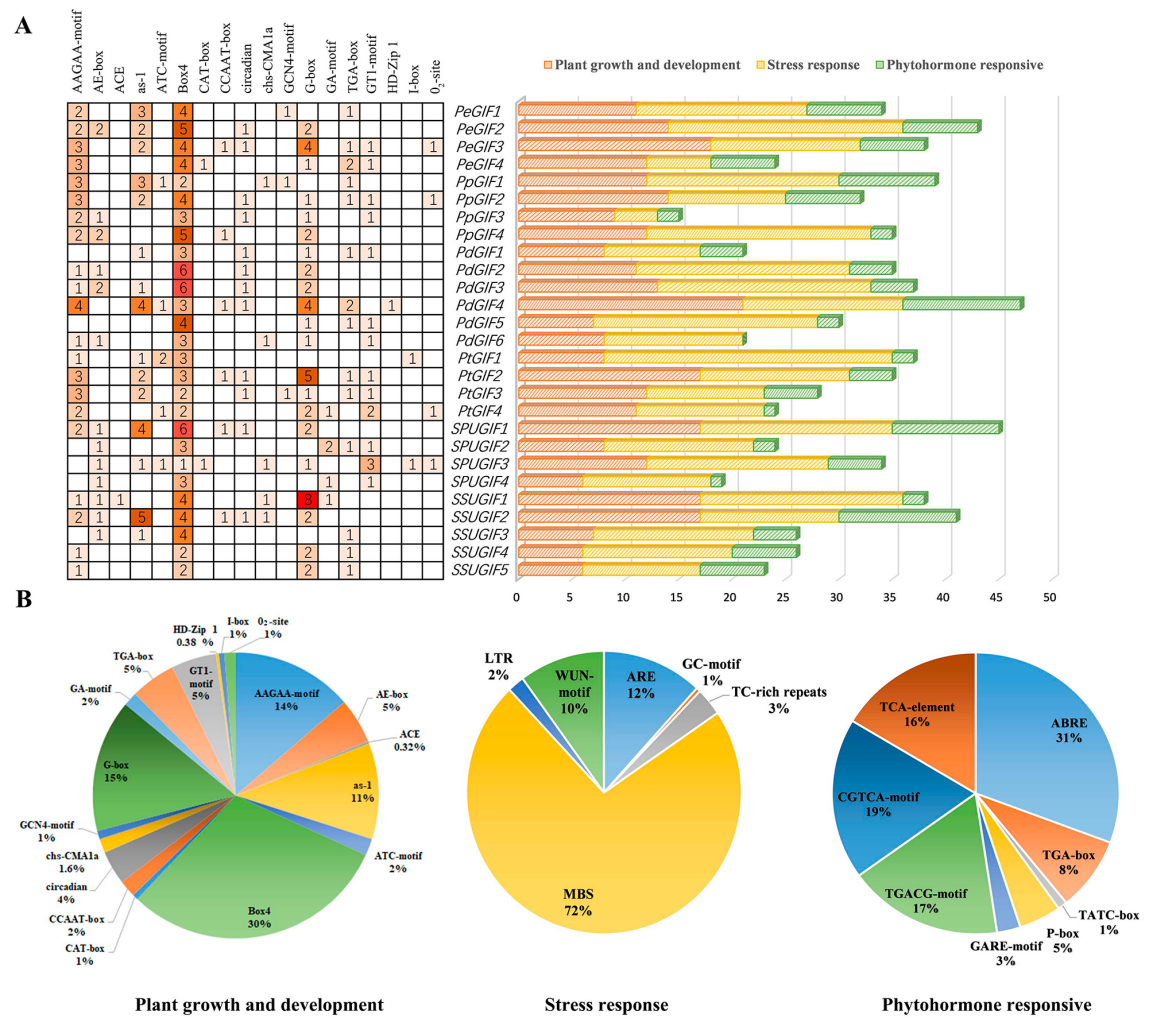
*Cis*-element analysis of *GIF* promoters in six Salicaceae species. **(A)** Numbers and gradient red colors serving as indicators of the abundance of *cis*-acting elements related to plant development present in each gene. **(B)** Color-coded histograms depicting the distribution of *cis*-acting elements in each gene, categorized into three distinct groups. **(C)** Pie charts depicting the distribution of distinct *cis*-acting elements within each category

The PlantCARE online website was used to predict *cis*-acting elements to analyze the possible factors influencing the expression patterns of *GIF* gene family members. The promoters of almost all *GIF*s contained various *cis*-acting elements associated with plant growth and development, phytohormone responses and stress responses (Fig. 4B). For instance, elements related to plant development, such as CAT-box, TGA-box, AAGAA-motif, GCN4-motif, as-1, Box4, G-box and so on (Fig. 4A). Meanwhile, stress-related elements are regulated by specific *cis*-acting motifs including antioxidant response element (ARE), GC-rich motif (GC-motif), TC-rich repeats (TCRRs), MYB binding site (MBS), low temperature-responsive (LTR) element, and wound-induced promoter motif (WUN-motif). The presence of MBS associated with drought stress response was observed in all *GIFs*, indicating their crucial role in mediating the response to drought-induced stress. Among the hormone-responsive elements, the prominent ones included abscisic acid (ABRE), auxin (TGA-element), gibberellin (TATC-box, P-box, and GARE-motif), MeJA (TGACG-motif and CGTCA-motif), and salicylic acid (TCA-element). The *cis*-acting elements related to MeJA were the most prevalent among hormone-responsive elements. The analysis suggested that *GIF* genes may participate in diverse growth and developmental processes possibly mediated by hormone signal transduction or environmental stimulus.

### Expression of *PeGIFs* in heteromorphic leaves from juvenile to adult

**Fig. 5:**
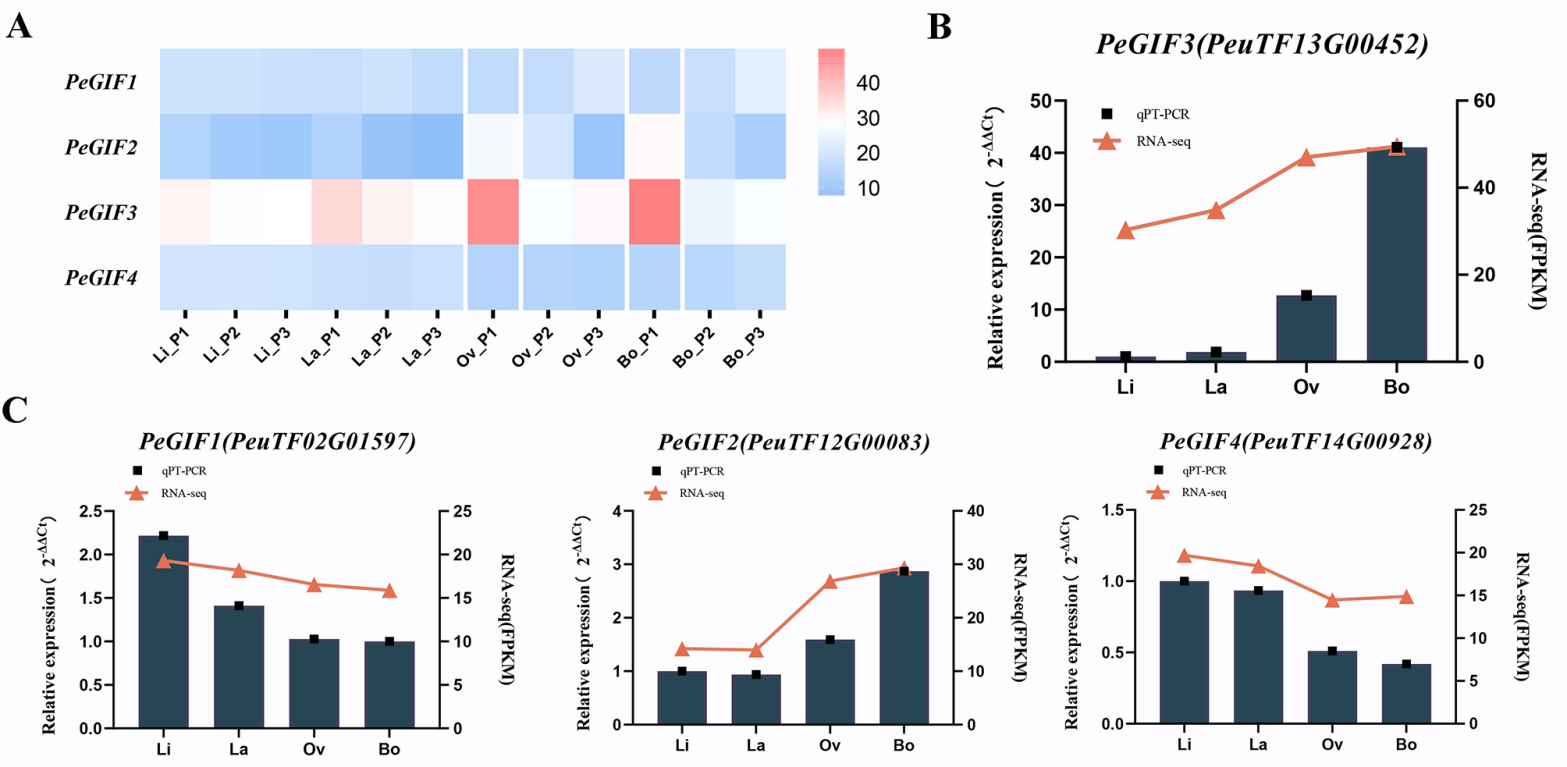
*PeGIF* genes expression patterns in heteromorphic leaves. **(A)** Expression patterns of *PeGIFs* across three stages (P1, P2, and P3) in four heteromorphic leaves (Li, La, Ov, and Bo). **(B)** qRT-PCR data on the expression patterns of *PeGIF3* for heteromorphic leaves in P1 stage. **(C)** qRT-PCR data on the expression patterns of other *PeGIFs* for heteromorphic leaves in P1 stage

The expression levels on four types of heteromorphic leaves (Li, La, Ov, and Bo) at three different developmental stages (P1, P2, and P3) were assessed to investigate the transcriptional regulation of *PeGIFs* underlying the developmental and functional differentiation processes of heteromorphic leaves. The expression of PeuTF02G01597 (*PeGIF1*), and PeuTF14G00928 (*PeGIF4*) exhibited very similar in four leaf shapes and did not exhibit regular changes. By contrast, the expression of PeuTF13G00452 (*PeGIF3*) in leaves with different leaf shapes increased continuously during the same period, and it was specifically upregulated in Ov and Bo leaves at the early stage of leaf development (Fig. 5A). *PeGIF3* showed regular changes in different leaf shapes and its expression level was significantly higher than that of PeuTF12G00083 (*PeGIF2*). *PeGIF3*, which is homologous to *ATGIF1* in *A. thaliana*, which plays a crucial role in leaf development. Moreover, the expression of *PeGIF3* significantly decreased during the later stages of leaf growth (P2 and P3), suggesting its potential involvement in promoting broad-leaf expansion during early morphogenesis. These results suggested that *PeGIF3* plays a dynamic role in regulating the development of broad leaves (Ov and Bo) in *P. euphratica*, qRT-PCR analysis was further adopted to confirm the expression levels of *PeGIFs* in heteromorphic leaves at P1 (Fig. 5B∼5 C). All the *PeGIFs* evaluated by qRT-PCR showed a similar expression pattern from RNA-seq results. Collectively, *PeGIF3* also encompasses multiple growth-related elements (AAGAA-motif and as-1), particularly auxin-related elements (TGA-box). These results indicated that up-regulation of *PeGIF3* may regulate the occurrence of broad heteromorphic leaves in *P. euphratica*.

### Subcellular localization of *PeGIF3*

**Fig. 6:**
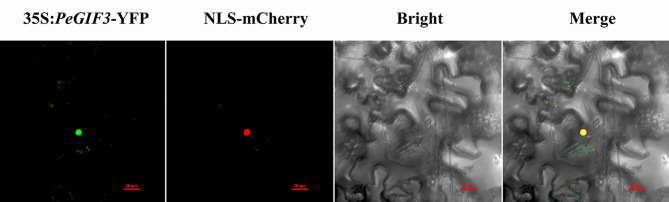
Nuclear localization of 35 S: *PeGIF3*-YFP protein in tobacco leaf epidermal cells: fluorescent images of *PeGIF3* (35 S:*PeGIF3*-YFP), nuclear localization signal (NLS-mCherry), and merged images (35 S:*PeGIF3*-YFP/NLS-mCherry). Bar = 20 μm

The GV3101 *Agrobacterium* strain carrying the 35 S:*PeGIF3*-YFP construct was introduced into tobacco (*Nicotiana benthamiana*) to investigate the subcellular distribution of the PeGIF3 protein in plant cells, and the subcellular localization of *PeGIF3* was visualized using a confocal laser scanning microscope. The fluorescence signal of 35 S: *PeGIF3*-YFP coincides with the nuclear localization signal of NLS-mCherry (Fig. 6). The subcellular localization of *PeGIF3* was observed to be predominant in the nucleus.

### Function of *PeGIF3* involving leaf phenotypes

**Fig. 7:**
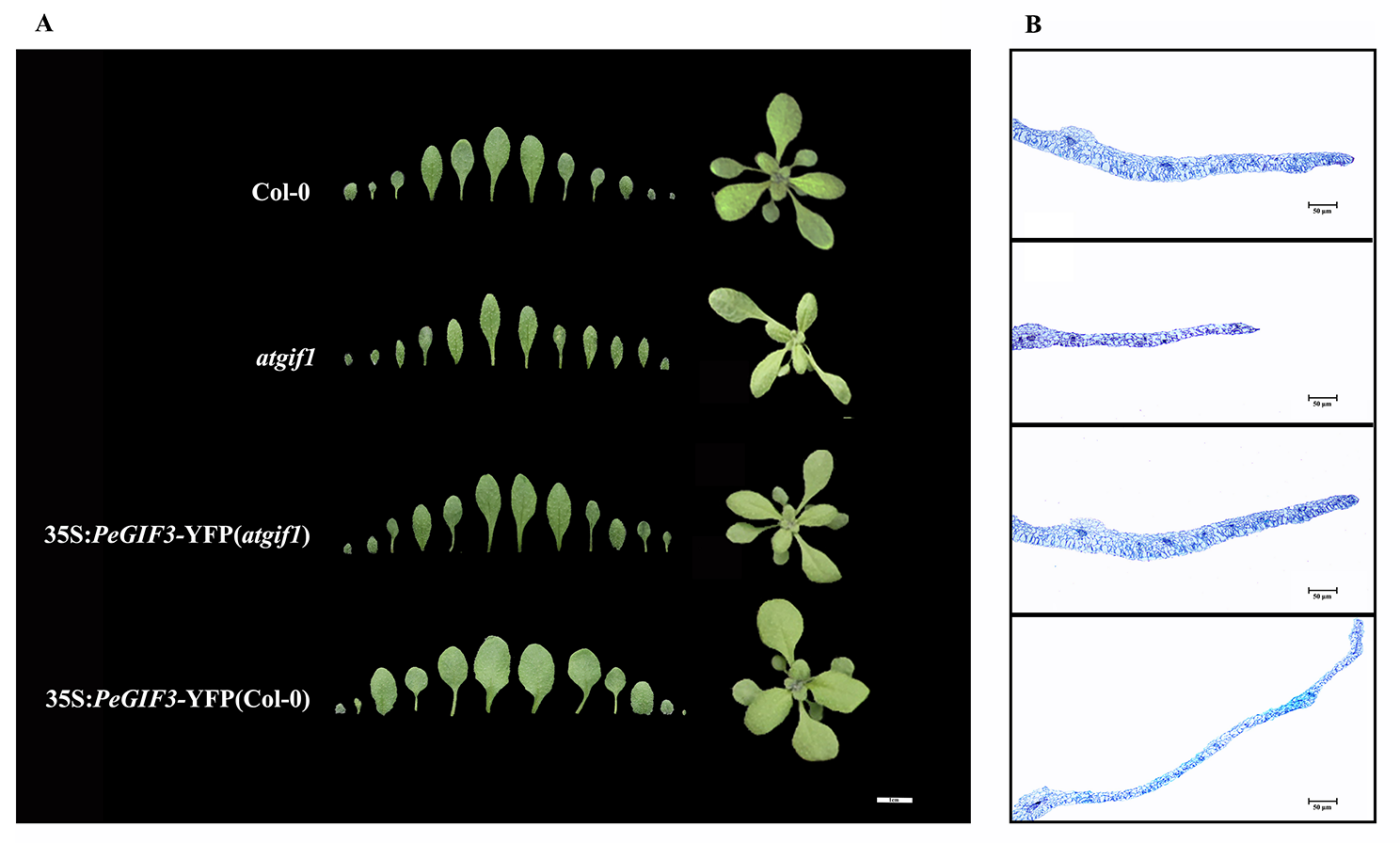
*PeGIF3* gene regulates leaf morphology. **(A)** The overall morphological characteristics of various plant species and morphological of rosette leaf in different plant lines. Bar = 1 cm. **(B)** Cross-sectional anatomical structure of the fully expanded first rosette leaf in each plant. Bar = 50 μm

Fully expanded first rosette leaf was derived from the 10-day-old wild-type plants, *atgif1* mutant plants, and transgenic wild-type plants overexpressing the *PeGIF3* gene, and the complementation of *PeGIF3* in *atgif1* mutant plants were analyzed to elucidate the cellular underpinnings of transgenic plant phenotypes. Twenty specimens of each line were selected for sampling. The *atgif1* mutant exhibited a more pronounced leaf width and leaf area defect. On the contrary, leaf width and leaf area were significantly higher in the overexpressed transgenic plants than in the wild-type plants (Fig. 7A). However, no significant differences were found in leaf length (Table 2). The phenotypes of transgenic *atgif1* plants expressing the *PeGIF3* gene were comparable to those of the wild type. Subsequently, we conducted analysis of leaf anatomy and observed significant variations in cell number among plants with different backgrounds (Fig. 7B). However, no statistically significant differences were detected in terms of cell area. The findings suggest that *PeGIF3* promotes leaf cell proliferation, leading to the expansion of the central-lateral region of the leaf, potentially enhancing leaf area.

**Table 2:** Effects of *PeGIF3* on leaf morphology changes

Genotype	Leaf area (mm^2^)	Leaf length (mm)	Leaf width (mm)	Leaf index^b^	Cell area (µm^2^)	Cell number
Col-0	378.9 ± 0.12	8.25 ± 0.16	5.19 ± 0.09	1.59 ± 0.19	76.08 ± 15.93	351.33 ± 34.89
*atgif1*	149.6 ± 0.05 a	8.31 ± 0.22	3.28 ± 0.15 a	2.53 ± 0.50 a	80.33 ± 19.18	125.51 ± 10.73 a
35 S:*PeGIF3*-YFP (*atgif1*)	354.3 ± 0.51	8.16 ± 0.18	5.03 ± 0.31	1.62 ± 0.31	81.54 ± 13.28	343.25 ± 35.01
35 S:*PeGIF3*-YFP (Col-0)	862.1 ± 0.31 a	8.21 ± 0.31	8.07 ± 0.05 a	1.01 ± 0.05 a	74.95 ± 6.51	744.52 ± 25.17 a

Fully expanded first rosette leaf of the 10-day-old plants were selected for measurement

a Statistically significant differences were observed compared to the wild type (*P* < 0.05)

b Proportion between the leaf blade’s length and width

## Discussion

The heteromorphic leaves in *P. euphratica* exhibit functional divergence at physiological and cytological levels. GIF proteins, recognized as key players in leaf development, positively regulate leaf size. Previous investigations predominantly focused on the involvement of *GIF* genes in plant growth and development, including their role in maintaining the homeostasis of leaf, seed, and root meristems in *A. thaliana* [14, 18, 25]. *GIF* genes have also been associated with modulating tissue and organ size in rice [19, 26, 27]. In maize, they have been implicated in regulating shoot architecture and meristem determinacy [21]. However, their presence and characteristics in Salicaceae remain unexplored. Recent releases of high-quality genomes for Salicaceae species, including *P. euphratica*, *P. pruinose, P. deltoides*, *P. trichocarpa*, *S. sinopurpurea*, and *S. suchowensis*, have opened new avenues for studying the *GIF* family in Salicaceae. In the present study, a total of 27 members belonging to the *GIF* family were identified within six Salicaceae species. Four genes were identified in each of the following species: *P. euphratica*, *P. pruinosa*, *P. deltoides*, and *S. sinopurpurea*. Five genes were identified in *P. deltoides* and *S.suchowensis*, allowing for an exploration of their structures and phylogenetic relationships within these species. The 27 *GIF* members were classified into three subfamilies on the basis of domain analysis and phylogenetic tree to confirm the evolutionary relationships between *GIFs*. The presence of highly conserved domains in the GIF protein within the same group suggested a potential similarity in function. The *ATGIF1* transcription coactivator gene was previously characterized as a positive regulator of cell proliferation in lateral organs, such as leaves and flowers, of *A. thaliana* [16]. In the present study, members located in the same subfamily as *ATGIF1* were hypothesized to function in regulating plant growth.

The presence of the highly-conserved SSXT motif was detected in all members comprising the *GIF* gene family. The findings are in line with those of prior research [11]. The N-terminal region of GIF proteins shares similarity with the SNH domain discovered in SYNOVIAL TRANSLOCATION (SYT) in humans, which interacts with BRAHMA (BRM) and BRAHMA RELATED GENE1 (BRG1), two ATPases involved in SWITCH/SUCROSE NONFERMENTING (SWI/SNF) chromatin remodeling processes in human cells. Based on sequence similarity, GIF transcriptional coactivators may act together by reciprocally binding to SWI/SNF chromatin remodelers [28]. Motifs 2 and 5 at C-terminus were presented in the *GIF* members of Salicaceae species only, implying that these members may have undergone functional divergence or acquired novel functions throughout the course of plant evolution.

The promoter analysis unveiled a significant number of plant growth and development elements (CAT-box, TGA-box, AAGAA-motif, GCN4-motif, as-1, Box4, and G-box ), phytohormone-responsive elements (ABRE, CGTCA, and TGACG), and stress-responsive elements (ARE, MYB, and MYC) in the promoter regions of the *GIF* genes. Among these elements, all genes have Box4 elements, which are part of the conserved DNA module involved in light response. In addition, G-box were important for early senescence of rice flag leaves [29]. Notably, auxin-related elements (TGA-box) were present in most members of each species, suggesting a role for *GIF* genes in regulating *Populus* growth and development. Other elements related to growth and development in some *GIF* genes were found, including the CAT-box related to meristem expression and the GCN4-motif related to endosperm expression. These *GIF* gene members have been linked to plant development, a finding consistent with that of previous research in *A. thaliana* [30].

The analysis of gene expression patterns in heteromorphic leaves led to the identification of differentially regulated genes specific to *P. euphratica*. The expression of *PeGIF3* significantly increased and exhibited a notable disparity between the heteromorphic leaves at P1, which is consistent with the qRT-PCR results. Therefore, *PeGIF3* may be closely regulated in increasing the size of broad leaves early during leaf morphogenesis in *P. euphratica*. The examination of transgenic plants showed a significant difference in the number of leaf cells between the overexpressed plants and the wild type. The subsequent observation revealed that *PeGIF3* was predominantly observed in the nucleus, consistent with the conserved motifs analysis of *GIFs* in multi-species in this study. Therefore, *PeGIF3* may enhance leaf cell proliferation, thereby resulting in the expansion of the leaf central-lateral region.

## Conclusion

In this study, 27 *GIF* genes were identified in six Salicaceae species, and their structures, phylogenetic relationships, conserved motifs and collinearity across Salicaceae species were characterized, with *Arabidopsis* as an outgroup. The detailed *cis*-element analysis showed that the Salicaceae *GIFs* are involved in multiple developmental processes and regulated by diverse factors such as phytohormones signals and environmental stimulus. Only *PeGIF3* showed a gradual upregulation along with the development of heteromorphic leaves of Li, La, Ov, and Bo, and it was specifically upregulated in Ov and Bo leaves at the early stage of leaf development. The essential involvement of *PeGIF3* in *P. euphratica* leaf development was elucidated using RNA-Seq data and qRT-PCR. Further overexpression of *PeGIF3* in the *atgif1* mutant and wild type of *Arabidopsis* results in enhanced leaf expansion along the medial-lateral region and increased cell population. The findings provide a foundation for further functional investigations into *GIF* genes in Salicaceae species and promote the study on leaf morphological variation among these species.

## Methods

### Identification and characterization of *GIF* homologs in salicaceae

The *PeGIFs* were identified on the basis of *P. euphratica* genome data [31]. The hidden Markov model (HMM) profiles for the GIF domain SSXT (PF05030) were acquired from the Pfam protein family database (http://pfam.xfam.org). HMMER (version 3.0, http://hmmer.org/) was employed to conduct a search for potential *GIF* genes in the six Salicaceae species. The superfluous candidate genes were excluded, and the remaining genes underwent additional validation using SMART (http://smart.emblheidelberg.de/). The protein physicochemical properties of GIF proteins, such as the amino acid count, molecular weight, and theoretical isoelectric point, were determined using the ProtParam tool (http://web.expasy.org/protparam/). The *GIFs* from five other Salicaceae species were identified on the basis of the genome data of *Populus pruinose* (National Center for Biotechnology Information, with BioProject accession number PRJNA863418), *P. deltoides* (WV94_445) [32], *P. trichocarpa* (V3.1) [4], *S. sinopurpurea* [33], and *S. suchowensis* [34]. The chromosomal location of *GIFs* was obtained from the genome annotation files, and the chromosome physical location of the *GIF* genes was displayed using MapChart software (version 2.32).

### Analysis of phylogenetic relationship consensus sequence in multi-species *GIFs*

A phylogenetic tree was constructed using the amino acid sequences that encode *GIF* genes from *P. euphratica* and various other species. The SMART website was utilized to extract the domain coordinates from the GIF protein sequence of *P. euphratica* and other various species. The sequences of the GIF domain were extracted using its coordinates and merged into a new sequence matrix. Then, the merged protein sequences were aligned by ClustalW. After the amino acid sequences were aligned, gap trimming was performed using the multiple alignment trimming tools of TBtools software [35], with a site coverage cutoff parameter set at 0.95. Subsequently, a phylogenetic tree was constructed using MEGA v7 software employing the neighbor-joining (NJ) method with 1000 bootstrap replicates. The percentage of replicate trees in which the associated taxa clustered together in the bootstrap test (1000 replicates) was shown next to the branches. The Dayhoff matrix-based method was used to calculate evolutionary distances, which were expressed as the number of amino acid substitutions per site. Ambiguous positions were excluded for each pair of sequences using the pairwise deletion option. TBtools and iTOL online website (https://itol.embl.de/) were used to visualize the phylogenetic tree.

Additionally, we used the MEME tool (http://meme-suite.org/) to classify and analyze the conserved motifs of each GIF protein sequence. We set the maximum motif number was 5 and other parameters are default settings.

### Collinearity analysis of multi-species *GIFs*

The BLASTP alignment was used to identify orthologous pairs between *P. euphratica* and six other species (*P. pruinose, P. trichocarpa, P. deltoides, S. sinopurpurea, S. suchowensis*, and *A. thaliana*). Then, the collinear blocks between *P. euphratica* and each other species of *P. deltoide*, *P. trichocarpa*, *A. thaliana*, *S. sinopurpurea* and *S. sinopurpurea* were identified using MCscan software and visualized using JCVI (https://zenodo.org/record/31631/).

**Promoter analysis of*GIF*promoters**.

The upstream 2000 bp (bp) sequences apart from the transcription start sites of these *PeGIFs* genes were identified as potential promoters using TBtools. Subsequently, the *cis*-elements within each promoter were identified using PlantCARE (http://bioinformatics.psb.ugent.be/webtools/plantcare/html/).

### RNA-seq for heteromorphic leaves

A total of 12 samples for four leaf shapes in cultivated forests, including Li, La, Ov, and Bo leaves, were collected across the development of leaf age. These samples were collected at various stages of leaf development. Leaf age was categorized into three periods based on field sampling and observation. The P1 was defined as the first day when the leaf blades started unfolding. This was followed by P2 occurring on the 15th day when there was an increase in leaf area. Finally, P3 occurred on the 30th day when leaves reached maturity. Each type of heteromorphic leaves with different leaf ages was replicated three times for sampling. The napkin was used to delicately clean the leaves, which were then rapidly frozen in liquid nitrogen and stored at an ultra-low temperature of -80℃ in a refrigerator for RNA-seq analysis (the dataset has been made available to the public for access [7] and preservation through the National Genomics Data Center (https://ngdc.cncb.ac.cn/), under project number PRJCA005959.

### Analysis of transcriptomes using short reads from illumina sequencing

As part of the study, we conducted whole transcriptome sequencing using mRNA-Seq on an Illumina Hiseq X-Ten platform, following the protocol recommended by the vendor. To assess the relative abundance of the annotated genes from *P. euphratica*, we employed HISAT2 (version 2.0.4) [36] to align the clean reads against our reference genome. The gene expression was quantified with FPKM using StringTie [37].

### Validation of *PeGIFs* using quantitative reverse-transcription polymerase chain reaction (qRT-PCR)

The heteromorphic foliage was collected from different canopies and stored in an ultra-low temperature refrigerator at -80℃ after being rapidly frozen with liquid nitrogen. The procedure followed the methodology described in a previous publication [38]. *Actin* gene was used as the endogenous control. Each reaction was performed in biological triplicates, and CT values obtained through qRT-PCR were analyzed using the 2^−ΔΔCT^ method to calculate relative fold change values.

### Plant growth conditions, treatments, and sampling

All *A. thaliana* mutants and transgenic plants that were used in this study were from the Columbia (Col-0) ecotype. The *Arabidopsis* seeds were sown on moist soil, stratified at 4℃ for 3 days, and then transferred to a growth room with a temperature of 21℃ and a photoperiod of 16 h light/8 hours darkness. *Atgif1* (SALK_208834C) seeds were obtained from the AraShare (https://www.arashare.cn/index/).

The leaves of *P. euphratica* were collected from the forest located at the eastern entrance of Tarim University.

### Cloning, construction of transgenic plants

The laboratory has preserved *Escherichia coli* (DH5α), *Agrobacterium tumefaciens* (GV3101), overexpressed vector (pGreenII 0179). The RNA was extracted from *P. euphratica* Bo leaves using Trizol (Invitrogen, Co.,Ltd), followed by cDNA synthesis using the M5 Sprint qPCR RT kit with gDNA remover (Mei5 Biotechnology, Co.,Ltd). The full-length coding regions of *PeGIF3* genes lacking a stop codon were amplified from cDNA, or plasmid using Phanta Max Super-Fidelity DNA polymerase (Vazyme, Co.,Ltd) to ensure high fidelity. Subsequently introduced into a yellow fluorescent protein (YFP) vector to generate a construct using the T4 DNA Ligase (Sangon Biotech, Co.,Ltd). The Pe*GIF3*::YFP fusion was inserted into the pGreenII 0179 vector, which contained a CaMV 35 S promoter and a NOS terminator cassette. The floral dip method was utilized for the transformation of *Arabidopsis* plants [39, 40]. The overexpression of *PeGIF3* was established with a wild-type background. The single-insertion homozygous T3 lines of the *PeGIF3* complement were carefully chosen and established in the *atgif1* mutant background. Three lines were selected for analysis in each transgenic plants with different backgrounds.

### Subcellular localization of *PeGIF3* gene

Transform the constructed 35 S:*PeGIF3*-YFP vector into *Agrobacterium tumefaciens* GV3101. Reconstitute the strain harboring the target plasmid (NLS-mcherry) in LB medium supplemented with appropriate antibiotics for overnight cultivation. Inoculate the bacterial solution obtained in the second step into fresh LB medium, simultaneously adding acetosyringone, and agitate until the bacteria reach an optical density (OD600) of 1.0-1.2. The supernatant should be discarded by centrifugation, and the bacteria should be resuspended in infection fluid (0.01 M MES (pH = 5.6), 0.01 M MgCl_2_·6H_2_O and 50 µM acetosyringone) until the OD value reaches approximately 1.0. Allow it to remain undisturbed for a duration of 3 h in a lightless environment. The target bacterial was combined with the NLS-mcherry in equal proportions, and tobacco were inoculated using a syringe. The treated plants were kept in darkness for 12 h and subsequently incubated under normal conditions for 36 h. The underlying epidermis of tobaccowas revealed in a dark environment and examined using a laser scanning confocal microscope (Nikon eclipse Ti2). The microscope was excited by a 488 nm laser and emitted signals were detected within the range of 500–550 nm.

### Measurement of leaf phenotypes and cellular morphology

The leaf cross-section chosen for anatomical analysis was carefully selected to encompass the widest point of the primary vein and subsequently fixed using FAA solution. The paraffin section method was employed to convert these into permanent film [41]. The samples were subsequently examined and imaged using a scanning electron microscope (OPLENIC CORP). Cells present in the pericycle to the leaf margin were enumerated. The statistical analyses were conducted using Graphpad Prism 9 software. The least significant difference test was employed to determine statistically significant differences between means at a significance level of *p* < 0.05.

Leaf morphology and cell area were analyzed with an image analyzer (ImageJ Launcher-1.4.3.67).

### Electronic supplementary material

Below is the link to the electronic supplementary material.

Supplementary Material 1

## Data Availability

RNA-seq data for P. euphratica’s heteromorphic leaves used in this study have been submitted to the NGDC(National Genomics Data Center, https://ngdc.cncb.ac.cn/) under the BioProject accession number PRJCA005959.

## References

[1] Bradshaw H, Ceulemans R, Davis J, Stettler R (2000). Emerging model systems in plant biology: poplar (*Populus*) as a model forest tree. J Plant Growth Regul.

[2] Brunner AM, Busov VB, Strauss SH (2004). Poplar genome sequence: functional genomics in an ecologically dominant plant species. Trends Plant Sci.

[3] Taylor G (2002). Populus: *Arabidopsis* for forestry. Do we need a model tree?. Ann Botany.

[4] Tuskan GA, Difazio S, Jansson S, Bohlmann J, Grigoriev I, Hellsten U, Putnam N, Ralph S, Rombauts S, Salamov A (2006). The genome of black cottonwood, *Populus trichocarpa* (Torr. & Gray). Science.

[5] Qiu Q, Ma T, Hu Q, Liu B, Wu Y, Zhou H, Wang Q, Wang J, Liu J (2011). Genome-scale transcriptome analysis of the desert poplar, *Populus Euphratica*. Tree Physiol.

[6] Song Z, Ni X, Yao J, Wang F (2021). Progress in studying heteromorphic leaves in *Populus Euphratica*: leaf morphology, anatomical structure, development regulation and their ecological adaptation to arid environments. Plant Signal Behav.

[7] Wu Z, Jiang Z, Li Z, Jiao P, Zhai J, Liu S, Han X, Zhang S, Sun J, Gai Z (2023). Multi-omics analysis reveals spatiotemporal regulation and function of heteromorphic leaves in Populus. Plant Physiol.

[8] Liu Y, Li X, Chen G, Li M, Liu M, Liu D (2015). Epidermal micromorphology and mesophyll structure of *Populus Euphratica* heteromorphic leaves at different development stages. PLoS ONE.

[9] Hao J, Yue N, Zheng C (2017). Analysis of changes in anatomical characteristics and physiologic features of heteromorphic leaves in a desert tree, *Populus Euphratica*. Acta Physiol Plant.

[10] Kim JH, Tsukaya H (2015). Regulation of plant growth and development by the *GROWTH-REGULATING FACTOR* and *GRF-INTERACTING FACTOR* duo. J Exp Bot.

[11] Kim JH, Kende H (2004). A transcriptional coactivator, *AtGIF1*, is involved in regulating leaf growth and morphology in *Arabidopsis*. Proc Natl Acad Sci U S A.

[12] Horiguchi G, Kim GT, Tsukaya H (2005). The transcription factor *AtGRF5* and the transcription coactivator *AN3* regulate cell proliferation in leaf primordia of *Arabidopsis thaliana*. Plant Journal: Cell Mol Biology.

[13] Vercruyssen L, Verkest A, Gonzalez N, Heyndrickx KS, Eeckhout D, Han SK, Jégu T, Archacki R, Van Leene J, Andriankaja M (2014). *ANGUSTIFOLIA3* binds to SWI/SNF chromatin remodeling complexes to regulate transcription during *Arabidopsis* leaf development. Plant Cell.

[14] Ercoli MF, Ferela A, Debernardi JM, Perrone AP, Rodriguez RE, Palatnik JF (2018). *GIF* Transcriptional Coregulators Control Root Meristem Homeostasis. Plant Cell.

[15] Li J, Pan W, Zhang S, Ma G, Li A, Zhang H, Liu L. A rapid and highly efficient sorghum transformation strategy using GRF4-GIF1/ternary vector system. Plant J 2023. 10.1111/tpj.16575 38038993

[16] Lee BH, Ko JH, Lee S, Lee Y, Pak JH, Kim JH (2009). The Arabidopsis *GRF-INTERACTING FACTOR* gene family performs an overlapping function in determining organ size as well as multiple developmental properties. Plant Physiol.

[17] Liu Y, Guo P, Wang J, Xu ZY (2023). Growth-regulating factors: conserved and divergent roles in plant growth and development and potential value for crop improvement. Plant J.

[18] Liang G, He H, Li Y, Wang F, Yu D (2014). Molecular mechanism of microRNA396 mediating pistil development in *Arabidopsis*. Plant Physiol.

[19] Duan P, Ni S, Wang J, Zhang B, Xu R, Wang Y, Chen H, Zhu X, Li Y (2015). Regulation of *OsGRF4* by *OsmiR396* controls grain size and yield in rice. Nat Plants.

[20] Qin L, Chen H, Wu Q, Wang X (2022). Identification and exploration of the GRF and GIF families in maize and foxtail millet. Physiol Mol Biology Plants.

[21] Zhang D, Sun W, Singh R, Zheng Y, Cao Z, Li M, Lunde C, Hake S, Zhang Z (2018). *GRF-interacting factor1* regulates shoot Architecture and Meristem Determinacy in Maize. Plant Cell.

[22] Ai G, Zhang D, Huang R, Zhang S, Li W, Ahiakpa JK, Zhang J. Genome-wide identification and molecular characterization of the *growth-regulating factors-interacting factor* Gene Family in Tomato. Genes 2020, 11(12). 10.3390/genes11121435 PMC7760089 33260638

[23] Wu ZJ, Wang WL, Zhuang J (2017). Developmental processes and responses to hormonal stimuli in tea plant (Camellia sinensis) leaves are controlled by GRF and GIF gene families. Funct Integr Genom.

[24] Zhang ZS, Zeng QY, Liu YJ (2021). Frequent ploidy changes in Salicaceae indicates widespread sharing of the salicoid whole genome duplication by the relatives of Populus L. and Salix L. BMC Plant Biol.

[25] Debernardi JM, Mecchia MA, Vercruyssen L, Smaczniak C, Kaufmann K, Inze D, Rodriguez RE, Palatnik JF (2014). Post-transcriptional control of *GRF* transcription factors by microRNA miR396 and *GIF* co-activator affects leaf size and longevity. Plant Journal: Cell Mol Biology.

[26] Yan S, Zou G, Li S, Wang H, Liu H, Zhai G, Guo P, Song H, Yan C, Tao Y (2011). Seed size is determined by the combinations of the genes controlling different seed characteristics in rice. TAG Theoretical Appl Genet Theoretische und Angewandte Genetik.

[27] Li S, Gao F, Xie K, Zeng X, Cao Y, Zeng J, He Z, Ren Y, Li W, Deng Q (2016). The *OsmiR396c-OsGRF4-OsGIF1* regulatory module determines grain size and yield in rice. Plant Biotechnol J.

[28] Xie Y, Skytting B, Nilsson G, Grimer RJ, Mangham CD, Fisher C, Shipley J, Bjerkehagen B, Myklebost O, Larsson O (2002). The SYT-SSX1 fusion type of synovial sarcoma is associated with increased expression of cyclin A and D1. A link between t(X;18)(p11.2; q11.2) and the cell cycle machinery. Oncogene.

[29] Liu L, Xu W, Hu X, Liu H, Lin Y (2016). W-box and G-box elements play important roles in early senescence of rice flag leaf. Sci Rep.

[30] Nelissen H, Eeckhout D, Demuynck K, Persiau G, Walton A, van Bel M, Vervoort M, Candaele J, De Block J, Aesaert S (2015). Dynamic changes in *ANGUSTIFOLIA3* Complex Composition reveal a Growth Regulatory mechanism in the Maize Leaf. Plant Cell.

[31] Zhang Z, Chen Y, Zhang J, Ma X, Li Y, Li M, Wang D, Kang M, Wu H, Yang Y et al. Improved genome assembly provides new insights into genome evolution in a desert poplar (*Populus Euphratica*). Mol Ecol Resour 2020, 20(3). 10.1111/1755-0998.13142 32034885

[32] Xue L, Wu H, Chen Y, Li X, Hou J, Lu J, Wei S, Dai X, Olson MS, Liu J. Evidences for a role of two Y-specific genes in sex determination in *Populus deltoides*. Nat Commun. 2020;11(1):5893. 10.1038/s41467-020-19559-2 PMC7674411 33208755

[33] Zhou R, Macaya-Sanz D, Carlson CH, Schmutz J, Jenkins JW, Kudrna D, Sharma A, Sandor L, Shu S, Barry K (2020). A willow sex chromosome reveals convergent evolution of complex palindromic repeats. Genome Biol.

[34] Dai X, Hu Q, Cai Q, Feng K, Ye N, Tuskan GA, Milne R, Chen Y, Wan Z, Wang Z (2014). The willow genome and divergent evolution from poplar after the common genome duplication. Cell Res.

[35] Chen C, Chen H, Zhang Y, Thomas HR, Frank MH, He Y, Xia R (2020). TBtools: an integrative toolkit developed for interactive analyses of big biological data. Mol Plant.

[36] Pertea M, Kim D, Pertea GM, Leek JT, Salzberg SL (2016). Transcript-level expression analysis of RNA-seq experiments with HISAT, StringTie and Ballgown. Nat Protoc.

[37] Kovaka S, Zimin AV, Pertea GM, Razaghi R, Salzberg SL, Pertea M (2019). Transcriptome assembly from long-read RNA-seq alignments with StringTie2. Genome Biol.

[38] Liu C, Hao J, Qiu M, Pan J, He Y (2020). Genome-wide identification and expression analysis of the MYB transcription factor in Japanese plum (*Prunus salicina*). Genomics.

[39] Zhang X, Henriques R, Lin SS, Niu QW, Chua NH (2006). Agrobacterium-mediated transformation of *Arabidopsis* thaliana using the floral dip method. Nat Protoc.

[40] Folkers U, Kirik V, Schöbinger U, Falk S, Krishnakumar S, Pollock MA, Oppenheimer DG, Day I, Reddy AS, Jürgens G (2002). The cell morphogenesis gene *ANGUSTIFOLIA* encodes a CtBP/BARS-like protein and is involved in the control of the microtubule cytoskeleton. EMBO J.

[41] Tsukaya H, Naito S, Rédei GP, Komeda Y (1993). A new class of mutations in *Arabidopsis thaliana*, acaulis1, affecting the development of both inflorescences and leaves. Development.

