# Supplementary material for: Comparative genomic analysis of the Growth-Regulating Factors-Interacting Factors (GIFs) in six Salicaceae species and functional analysis of PeGIF3 reveals their regulatory role in Populus heteromorphic leaves
Source: BMC Genomics. 2024 Mar 28;25:317. doi: 10.1186/s12864-024-10221-5 (PMC10976704; doi:10.1186/s12864-024-10221-5)
Supplement: Supplementary file 1 — Supplementary Material 1 [file 12864_2024_10221_MOESM1_ESM.pdf]

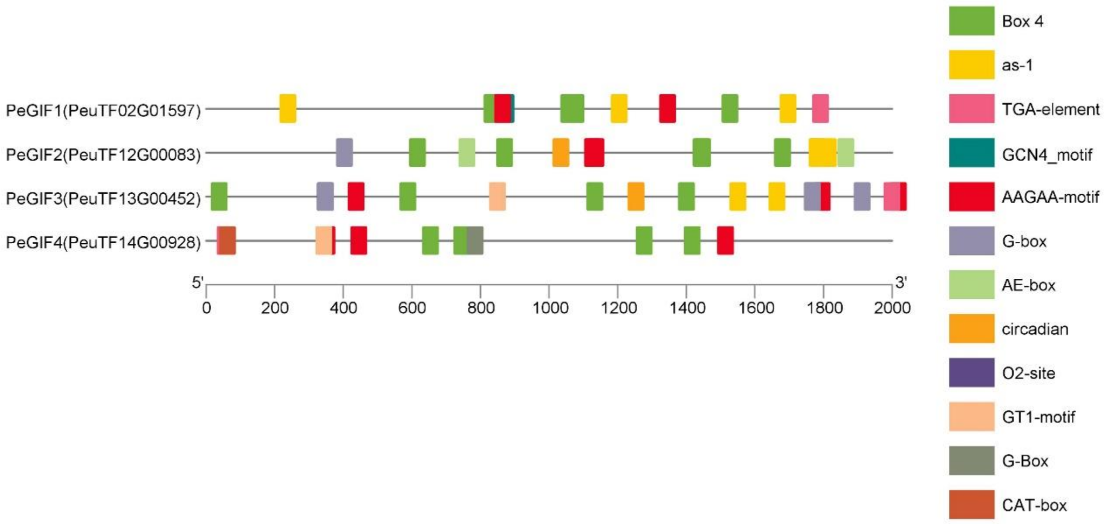

Figure S1. *Cis*-element analysis of *PeGIF3* promoters in regulating growth and development.

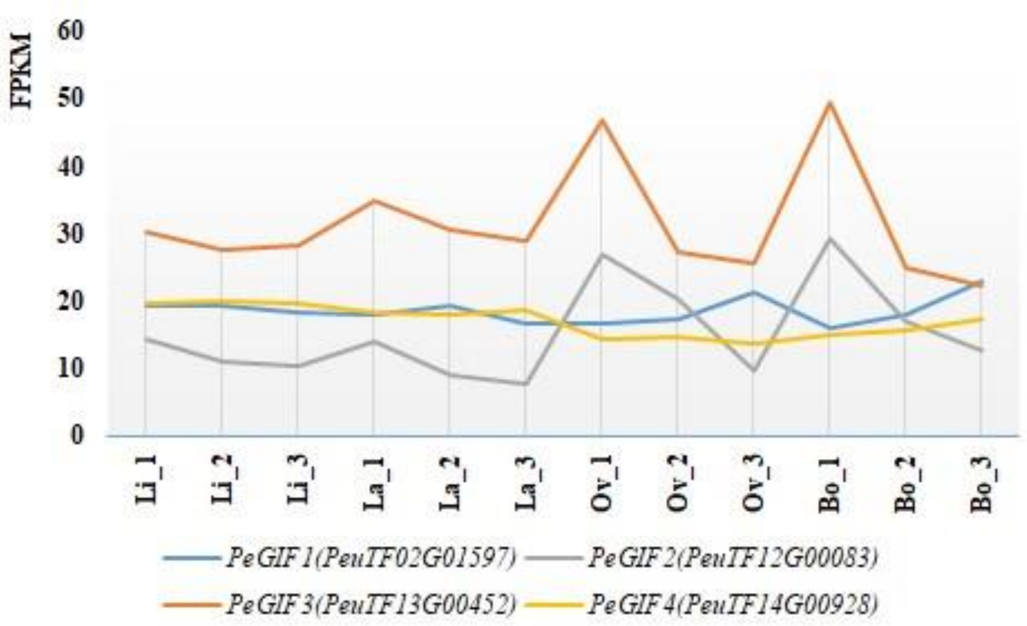

Figure S2. Expression patterns of *PeGIFs* across three stages in four heteromorphic leaves (Li, La, Ov and Bo).

Table S1. Primer sequences used in expression analysis of the *PeGIF* genes.

Gene	Forward and reverse primer sequence (5'~3')	Purpose
qPeGIF1-F	TTTCTTGCCACGAACCACAA	qRT-PCR
qPeGIF1-R	GTATCTGCCCGTGATAGGTGG	qRT-PCR
qPeGIF2-F	GTCACCGCAGCAAATGTTGA	qRT-PCR
qPeGIF2-R	GGTTCTGCTGTAACTGGGCT	qRT-PCR
qPeGIF3-F	GCAGCCAGGAGCACATTACA	qRT-PCR
qPeGIF3-R	TACTGCAGCATGGAAGAGCG	qRT-PCR
qPeGIF4-F	CCATCTCACAGGGTTAGGGC	qRT-PCR
qPeGIF4-R	TGGATCTGCTCAGTGGTGATG	qRT-PCR
PeActin-F	GTCCTCTTCCAGCCATCTC	qRT-PCR
PeActin-R	TTCGGTCAGCAATACCAGG	qRT-PCR
PeGIF3-F	ATGCAACAGCACCTGATGCAGATG	Cloning
PeGIF3-R	ATTCCCATCATCTGCAGATTTC	Cloning

All primers have no hairpin structure

Table S2. Transgenic plants of this study.

Name	Vector	Background
COM-1	35S:*PeGIF3*-YFP	*atgif1*
COM-2	35S:*PeGIF3*-YFP	*atgif1*
COM-3	35S:*PeGIF3*-YFP	*atgif1*
OE-1	35S:*PeGIF3*-YFP	Col-0
OE-2	35S:*PeGIF3*-YFP	Col-0
OE-3	35S:*PeGIF3*-YFP	Col-0
